# Disentangling Counter‐Empathy: Developing a Three‐Dimensional Model and Measure of Dispositional Counter‐Empathy

**DOI:** 10.1111/jopy.70023

**Published:** 2025-10-07

**Authors:** Jake R. Siamro, Christian H. Jordan

**Affiliations:** ^1^ Department of Psychology Wilfrid Laurier University Waterloo Ontario Canada

**Keywords:** affective sadism, aggression, counter‐empathy, dark triad, gluckschmerz, scale development, schadenfreude

## Abstract

**Objectives:**

Counter‐empathy involves responding to others' assumed emotions incongruently. Research on dispositional counter‐empathy predominantly focuses on specific counter‐empathic constructs without clearly mapping its cardinal dimensions. We develop and test a Three‐Dimensional Model of Counter‐Empathy (3DCE) that includes schadenfreude, gluckschmerz, and affective sadism.

**Method:**

Across five studies (total *N* = 1878), we test the 3DCE and develop the Various Indices of Counter‐Empathy (VICE). Study 1a and Study 1b administered items representing the 3DCE to develop the VICE. Study 2 administered the VICE, measures of counter‐empathic constructs, empathy, everyday sadism, and socially aversive outcomes. Study 3a and Study 3b administered vignettes of others' good fortunes and misfortunes, and depictions of general and social harms, and participants reported their reactions.

**Results:**

The 3DCE and validity of the VICE are supported by exploratory and confirmatory factor analyses; a “bass‐ackward” factor analysis mapping the hierarchical structure of counter‐empathy; incremental analyses predicting socially aversive outcomes beyond empathy; correlations with relevant constructs; and predicting counter‐empathic reactions to specific scenarios.

**Conclusions:**

The 3DCE and VICE can help situate prior research in the broader structure of counter‐empathy, help expand the study of vicarious emotion beyond empathy, and suggest counter‐empathy contributes to socially aversive outcomes beyond a lack of empathy.

What drives people to act cruelly towards others? It is widely held that cruelty emerges partly from a failure to empathize—an inability or unwillingness to vicariously share in the joys and miseries of others. Yet growing evidence suggests cruelty may arise not from an empathy deficit, but through an active enjoyment of others' suffering and resentment of their happiness. Counter‐empathy is the tendency to respond to others' assumed emotions in an incongruent manner (specifically *opposite in valence*), such as positive emotion in response to others' misfortune or negative emotion in response to others' good fortune. Some people may be particularly prone to such experiences; those who are may also be likely to go further and antagonize people who are suffering or try to undermine others' happiness. Specific experiences of counter‐empathy predict socially undesirable outcomes: Dispositional schadenfreude (enjoyment of others' misfortunes), for instance, predicts online trolling (Brubaker et al. 2021), aggression (Erzi 2020), and intergroup conflict (Hudson et al. 2019). Despite its social implications, scant research explores the structure of counter‐empathy as a disposition.

Counter‐empathy research predominantly focuses on specific forms of counter‐empathy rather than a broader construct. There have been few efforts to map the cardinal dimensions of counter‐empathy, compare these dimensions to empathy or other conceptually related constructs, or determine whether individuals prone to one form of counter‐empathy are also prone to others. It is important to clearly map the underlying dimensions of counter‐empathy because existing measures of counter‐empathic constructs, and often the constructs themselves, often reflect an unspecified blend of potentially more basic forms of counter‐empathy. Tall Poppy Syndrome—a desire for misfortune to befall highly successful people—for example, blends displeasure at others' good fortune (i.e., their success) and pleasure at their misfortune (Feather 2008). Some measures of schadenfreude include items that assess a desire for successful people to experience negative outcomes (Crysel and Webster 2018). Such constructs and measures may be problematic because they may limit understanding of the broader disposition, promote the use of inconsistent terminology, and limit the integration of results from different research areas.

In this paper, we introduce a Three‐Dimensional Model of Counter‐Empathy (3DCE)—that consists of schadenfreude, gluckschmerz, and affective sadism—and develop a corresponding measure of dispositional counter‐empathy, the Various Indices of Counter‐Empathy (VICE). Schadenfreude and gluckschmerz—enjoyment of others' negative outcomes and discomfort with others' positive outcomes—are passive manifestations of counter‐empathy. Affective sadism is a more active manifestation, reflecting a specific type of sadistic pleasure‐seeking that occurs in response to the perceived emotional state of others. It is the tendency to derive pleasure from undermining others' positive emotions (e.g., antagonizing someone who seems happy) and amplifying emotional distress (e.g., taunting someone who is already upset).

## 1 Towards a More Comprehensive Study of Vicarious Emotion

People who lack empathy have been expected, in prior work, to engage in more socially aversive behavior (Miller and Eisenberg 1988; Paulhus 2014), but dispositional empathy is an unexpectedly weak predictor of aggression (Vachon et al. 2014). One reason for this may be that measures of affective empathy capture feeling *congruent* vicarious emotions but not *incongruent* ones (cf. Vachon and Lynam 2016). Low scores on empathy measures seem to reflect apathy or indifference—a lack of emotional response—rather than counter‐empathy. We expect counter‐empathy to be a more robust predictor of socially aversive behavior than empathy or indifference.

Empathy and counter‐empathy appear to be distinct constructs that predict different outcomes and activate different brain regions (e.g., Jie et al. 2022; Takahashi et al. 2009; Vachon and Lynam 2016). Although those prone to counter‐empathy are less likely to experience empathy, we believe these constructs are non‐redundant—each representing distinct experiences that reflect important aspects of responses to others' emotions. Quantifying the degree of empirical overlap between empathy and counter‐empathy can meaningfully affect how they are studied, including decisions about when researchers should distinguish between the two and whether controlling for one while analyzing the other is warranted (Lawson and Robins 2021).

## 2 Current Approaches to Studying Counter‐Empathy

What are the basic dimensions of dispositional counter‐empathy, the cardinal ways individuals differ in their tendency to respond oppositely to others' emotions? Limited work exists to establish the theoretical groundwork necessary for developing a comprehensive understanding of counter‐empathy. However, although the specific study of counter‐empathy is relatively new, it builds on a foundation of established and distinct constructs that share counter‐empathic elements, many of which are supported by decades of research. Current approaches treat counter‐empathy, implicitly or explicitly, as unidimensional or bidimensional.

### 2.1 Unidimensional Counter‐Empathy

One unidimensional measure of counter‐empathy is a subscale of a broader dispositional empathy scale, the Affective and Cognitive Measure of Empathy (ACME; Vachon and Lynam 2016). Within the ACME, counter‐empathy (termed affective dissonance) is viewed as schadenfreude and scorn for others' emotional experiences but is assessed as a single dimension. Notably, by incorporating (reversed) counter‐empathy into an empathy measure, this approach implies that empathy and counter‐empathy lie on a single continuum. In contrast, we contend that empathy and counter‐empathy are distinct dispositional tendencies. Further, inspecting the items of the ACME reveals that it includes five schadenfreude items, two gluckschmerz items, and five items that appear to capture everyday sadism (e.g., “If I could get away with it there are some people that I would enjoy hurting”). Although affective dissonance is a useful addition to this empathy measure, as a measure of counter‐empathy, the subscale is imbalanced and may conflate a conceptually distinct construct (everyday sadism) with counter‐empathy.

### 2.2 Bidimensional Counter‐Empathy: Gluckschmerz or Envy?

Other times, researchers specify two counter‐empathic reactions. Though schadenfreude is almost invariably included as a form of counter‐empathy, different researchers specify a second dimension—capturing negative reactions to others' good fortunes—as either envy (e.g., Boecker et al. 2022; Takahashi et al. 2009) or gluckschmerz (e.g., Hudson et al. 2019; Smith and van Dijk 2018). Envy is a negative affective response to an upward social comparison, triggered when another person possesses an advantage or achievement one lacks (Van de Ven et al. 2009). Schadenfreude and envy are specified in the Social Comparison Model, for example, as distinct reactions to others' good fortunes and misfortunes based on appraisals of how desirable an event is for oneself and for the other person (Boecker et al. 2022). Envy in this view is a response to a domain‐specific social comparison where one desires another's outcome. This is true of other models of envy, though there is debate over whether envy necessitates desire and whether it is best understood as a unitary construct or as having two distinct manifestations: *malicious envy*, which involves hostility and a desire to diminish the envied person's advantage, and *benign envy*, which motivates self‐improvement and upward emulation (Van de Ven et al. 2009).

Gluckschmerz is a broader construct involving a negative response to another's good fortune that does not depend on direct social comparison or desire to achieve the other's outcome (Smith and van Dijk 2018). Envy is rooted in feelings of inferiority (Crusius et al. 2020), whereas gluckschmerz can be motivated by additional factors like disliking the target or viewing their good fortune as undeserved. Because gluckschmerz is broader, we focus on it as a dimension of counter‐empathy in the 3DCE, rather than envy. Nevertheless, in testing our model—and developing our measure—we empirically compare the distinctness of gluckschmerz and envy.

### 2.3 Affective Sadism: Active Counter‐Empathy

The 3DCE also specifies an active dimension of dispositional counter‐empathy: affective sadism, the tendency to amplify or prolong others' negative emotions and undermine their positive emotions. Including affective sadism in our model is consistent with the view that schadenfreude and gluckschmerz presuppose, “a hostile action tendency or hostile disposition” that inclines people to act on others' emotions (Smith and van Dijk 2018, 297). This mirrors models of empathy that suggest empathizing leads to prosocial behavior to reduce the distress of others (Batson 1987). Counter‐empathy may go beyond passive reactions (e.g., enjoying others' distress) to include active efforts to harm others as a means of deriving personal pleasure.

There are close conceptual connections between schadenfreude and everyday sadism, a disposition to derive pleasure from causing others' suffering (Buckels et al. 2013). Some measures of everyday sadism include items that appear to assess schadenfreude (e.g., “I enjoy seeing people hurt,” O'Meara et al. 2011; see Parton and Chester 2025). Similarly, as noted, the ACME‐affective dissonance subscale incorporates items that seem to assess everyday sadism. Although both everyday sadism and schadenfreude involve experiencing positive emotions in response to others' negative experiences, everyday sadism uniquely includes actively harming through aggression (Chester et al. 2019). The empirical overlap in the assessment of these constructs further suggests that people prone to counter‐empathy may have a hostile disposition that can encourage sadism, though we argue everyday sadism is not strictly counter‐empathic.

Everyday sadism is not a reaction to others' emotions. Those high in everyday sadism have reduced sensitivity to context and are likely to aggress regardless of whether a target is visibly distressed or happy (Chester et al. 2019). Counter‐empathy, by definition, is a response to another's emotional state. Accordingly, we conceptualize affective sadism as a specific, relatively narrow manifestation of sadism that is counter‐empathic in nature. Nevertheless, we empirically test the distinctiveness of everyday sadism and affective sadism.

## 3 The Current Research

Across five studies, we test the 3DCE and develop the VICE, a multi‐dimensional measure of dispositional counter‐empathy. This model and measure can help bring greater clarity to counter‐empathy research. As noted, many counter‐empathic constructs and measures may blend distinct elements of counter‐empathy, making it difficult to identify their specific contributions to outcomes or compare findings across different areas of research (e.g., Tall Poppy Syndrome or schadenfreude). In other cases, different terminology may be used to study constructs that are indistinguishable from one another. The Varieties of Sadistic Tendencies (VAST) scale, for example, has a subscale assessing vicarious sadism, defined as a disposition to enjoy simulated/fantasized harm (Buckels 2023), which is conceptually similar to schadenfreude (Parton and Chester 2025). Our model and empirical tests can help clarify whether these constructs are distinct and whether vicarious sadism aligns more closely with schadenfreude or everyday sadism. In testing our model and the VICE, we empirically examine the extent to which different measures of counter‐empathic constructs represent a blend of 3DCE dimensions.

Studies 1a and 1b aim to develop a multidimensional measure while testing a three‐factor structure of counter‐empathy. Study 2 cross‐validates the factor structure of the VICE and tests its relations to other measures of counter‐empathic constructs and related constructs (e.g., everyday sadism). It also tests the incremental validity of the VICE over empathy in predicting socially aversive traits and behaviors. A “bass‐ackward” factor analysis (Goldberg 2006) situates the 3DCE dimensions in the broader taxonomy of counter‐empathic constructs. Studies 3a and 3b test whether the VICE predicts situational emotional responses to others' positive or negative outcomes. Overall, these studies serve to test the 3DCE, differentiate empathy and counter‐empathy, compare gluckschmerz to envy, and affective sadism to everyday sadism. In doing so, we develop the first measures of dispositional gluckschmerz and affective sadism and test their relations to other counter‐empathic constructs and socially aversive outcomes.

## 4 Study 1a and Study 1b

Studies 1a and 1b develop an initial itemset to test the 3DCE and develop the VICE through exploratory factor analyses (EFAs). We initially conceived of two active dimensions of counter‐empathy that stem from schadenfreude (i.e., prolonging or amplifying others' negative emotion) and gluckschmerz (i.e., undermining others' positive emotion). Accordingly, we generated items to reflect these two aspects of affective sadism, as well as schadenfreude and gluckschmerz. Although we initially conceived of four dimensions of counter‐empathy, the two active dimensions failed to separate into distinct factors in Studies 1a and 1b, leading us to specify the 3DCE and conclude that individuals prone to undermining others' positive emotions are substantially the same individuals who are prone to amplifying their negative emotions.

### 4.1 Method

#### 4.1.1 Participants

##### 4.1.1.1 Study 1a

Canadian undergraduates (*N* = 454) participated in exchange for partial course credit. Using preregistered exclusion criteria, participants were removed for reporting their data should not be used (*n* = 16), having too much missing data (> 10% of items; *n* = 12), inattentive responding (i.e., invalid responses to Infrequency or Virtue scales; *n* = 56), and being straight‐liners (i.e., invariant responses to the Rosenberg Self‐Esteem Scale; *n* = 9). The final sample (*N* = 361; 17.2% male, 80.9% female, 1.7% non‐binary, and 0.3% other) ranged in age from 17 to 48 (*M* = 20.65, SD = 3.95) and identified their ethnicities as: White (61.2%), South Asian (16.9%), East Asian (5.8%), Middle Eastern (3.6%), Caribbean (2.8%), Latin, Central, or South American (2.8%), African (2.2%), Indigenous (0.6%), and other (4.2%).

##### 4.1.1.2 Study 1b

U.S. adults (*N* = 450) were recruited from CloudResearch Connect in exchange for 2.50 USD, using census matching to collect a sample demographically similar to the U.S. general population (Table S1). Participants were removed for duplicate IP addresses (*n* = 2), inattentive responding (*n* = 29), and being identified as straight‐liners (*n* = 3). The final sample (*N* = 416; 49.5% male, 50.5% female) ranged in age from 19 to 77 (*M* = 43.43, SD = 14.31), identifying their ethnicity as: White (88%), African (6.7%), Latin, Central, or South American (4.3%), Caribbean (0.7%), and other (0.2%). Study 1b followed the same preregistration as 1a. Supporting Information for all studies and the preregistration, data, code, and materials for Studies 1a and 1b are at: https://osf.io/uxr8b/.

#### 4.1.2 Procedure

Participants completed counter‐empathy and demographics items online. Items from the abbreviated Infrequency and Virtue scales of the Elemental Psychopathy Assessment were evenly interspersed throughout the survey to assess data quality. Recent recommendations for EFAs recommend 400 participants (Goretzko et al. 2021). In Study 1b, we oversampled; though in Study 1a we fell short of 400 participants due to a technical oversight.

#### 4.1.3 Measures

##### 4.1.3.1 Counter‐Empathy Item Pool

Participants rated their agreement with all items on a 7‐point scale from (1) *strongly disagree* to (7) *strongly agree*. A pool of 120 items was generated by the researchers based on recommendations that an initial itemset should contain two to three times more items than expected in the final itemset (Carpenter 2018). We aimed to have 10 items per subscale (based on initial expectations of four subscales). Readability analysis of the items using the Flesch–Kincaid index (Flesch 1948) reveals they correspond to a 7th grade reading level.

##### 4.1.3.2 Infrequency and Virtue Scales

Participants indicated agreement with 3 items each from Infrequency and Virtue scales of the Elemental Psychopathy Assessment (Lynam et al. 2011). These were used to identify inattentive responders.

### 4.2 Results and Discussion

EFAs were used to determine the optimal number of factors to retain (see Supporting Information for analyses of the suitability of the data for factor analysis), using principal axis factoring (PAF) as it is less prone to inflating the number of retained factors than principal components analyses (Costello and Osborne 2005) with a Promax rotation, as it is considered the most robust oblique rotation (Thompson 2004). EFAs were conducted iteratively on each sample separately to eliminate items that cross‐loaded on multiple factors, did not load on any factors, or did not load on the intended factor. The 10 best items for each retained factor were chosen from the common remaining items in both samples (Table 1). Scree plots (Figures S1 and S2), parallel analyses using the Monte Carlo PCA for Parallel Analysis program (Watkins 2006; 1000 simulations), and minimum average partial correlations (MAP; Velicer 1976) were used to determine the number of factors to retain. Analyses in both samples indicated three factors, reflecting schadenfreude, gluckschmerz, and affective sadism. The two hypothesized dimensions of active counter‐empathy did not separate, but 5 items of each type were retained in the affective sadism factor. Factor loadings ranged from 0.34 to 0.90 in both samples (Table S2). Results for both samples are thus consistent with the 3DCE.

**TABLE 1:** Items for the Various Indices of Counter‐Empathy (VICE).

Factor	Item
Schadenfreude	I like it when people in movies have bad endings I enjoy it when couples in movies end up breaking up It can be funny seeing someone face unreasonably bad luck in a game I love stories where the “hero” ends up failing It can be amusing hearing about someone losing big at a casino. It can be fun to watch “fail” videos It would be pretty funny to hear about someone suddenly getting diarrhea I find it entertaining when I see someone get splashed by a car driving through a puddle It can be satisfying when someone else gets fired I sometimes enjoy seeing people humiliate themselves
Gluckschmerz	11 When someone's social media post gets a ton of likes, I can't help but feel a bit sour 12 I feel a bit bothered when I see someone who is well put together 13 I sometimes feel a little out of sorts when someone else's success is celebrated 14 I feel bitter when others succeed 15 I hate when good things happen to other people 16 When a friend receives compliments, I feel secretly annoyed 17 Seeing other people's achievements makes me sour 18 I feel pain at the sight of another's good fortune 19 It ruins my mood when I hear about something great happening to someone else 20 Very successful people are repulsive
Affective Sadism	21 There is a certain pleasure in irritating someone on their worst day. 22 Sad people are easy targets, so I like to torture them. 23 I like to kick people when they're down. 24 When someone is angry, I like to get them more worked up. 25 I like to make insecure people feel worse about themselves. 26 I like to pick fights with people who seem really happy. 27 I see the satisfaction of others as an opportunity to dampen their spirits. 28 When someone is excited, I imagine ways I could spoil their fun. 29 I enjoy tormenting others when they are *too* happy. 30 I feel a sense of accomplishment when I can undermine someone's happiness.

Reliabilities and scale norms of the VICE appear in Table 2. Across all samples, schadenfreude, gluckschmerz and affective sadism correlate between 0.37 and 0.61 suggesting they are related but non‐redundant. The VICE and its subscales demonstrate good reliability.

**TABLE 2:** Correlations and descriptive statistics for the Various Indices of Counter‐Empathy (VICE).

	1	2	3	*M*	SD	Reliability (*α*)
Study 1a (*n* = 361 Canadian undergraduates)						
Schadenfreude	—	—	—	2.53	1.02	0.83
2 Gluckschmerz	0.50	—	—	1.98	0.97	0.90
3 Affective Sadism	0.60	0.59	—	1.26	0.45	0.87
4 VICE Total	0.86	0.85	0.79	1.92	0.69	0.92
Study 1b (*n* = 416 US adults)						
Schadenfreude	—	—	—	2.25	1.02	0.87
2 Gluckschmerz	0.58	—	—	1.81	0.92	0.93
3 Affective Sadism	0.50	0.61	—	1.33	0.58	0.94
4 VICE Total	0.86	0.87	0.77	1.80	0.71	0.94
Study 2 (*n* = 392 US adults)						
Schadenfreude	—	—	—	2.61	1.09	0.88
2 Gluckschmerz	0.38	—	—	1.87	0.97	0.95
3 Affective Sadism	0.50	0.57	—	1.38	0.59	0.94
4 VICE Total	0.82	0.80	0.79	1.95	0.71	0.93
Study 3a (*n* = 378 Canadian undergraduates)						
Schadenfreude	—	—	—	2.84	0.98	0.82
2 Gluckschmerz	0.40	—	—	2.01	0.99	0.92
3 Affective Sadism	0.37	0.49	—	1.29	0.60	0.94
4 VICE Total	0.79	0.83	0.72	2.05	0.67	0.91
Study 3b (*n* = 331 Canadian adults)						
Schadenfreude	—	—	—	3.08	1.09	0.84
2 Gluckschmerz	0.35	—	—	2.19	1.22	0.94
3 Affective Sadism	0.39	0.49	—	1.29	0.64	0.93
4 VICE Total	0.76	0.83	0.72	2.19	0.77	0.92

*Note:* All correlations are significant at a value of *p* ≤ 0.001.

## 5 Study 2

Study 2 further tests the VICE's factor structure and construct validity. In doing so, it further tests the 3DCE. We test how VICE subscales relate to other counter‐empathy measures and related constructs (e.g., empathy). We also test if counter‐empathy incrementally predicts socially aversive behavior and personalities beyond empathy. In addition, we conduct a “bass‐ackward” factor analysis (Goldberg 2006) to map the emergence of increasingly specific manifestations of counter‐empathy using a broad sample of items from measures of counter‐empathic constructs; this tests the 3DCE as other manifestations could emerge before or instead of those indicated in our model. These analyses can enhance understanding of how counter‐empathic constructs interrelate and how they combine to form superordinate constructs.

### 5.1 Method

#### 5.1.1 Participants

U.S. adults (*N* = 452) were recruited from CloudResearch Connect in exchange for 5.25 USD, using census matching (Table S3). Participants were excluded for duplicate IP addresses (*n* = 4), reporting their data should not be used (*n* = 1), inattentive responding (*n* = 30), and being identified as straight‐liners (*n* = 25). The final sample consisted of 392 participants (48.5% male, 51.5% female), ranging in age from 18 to 85 (*M* = 45.78, SD = 14.36), identifying their ethnicity as: White (86.7%), African (7.4%), Latin, Central, or South American (3.1%), Caribbean (0.8%), Indigenous (0.3%), South Asian (0.3%), and other (1.5%). Data, analysis code, and materials are at: https://osf.io/5xghy/

#### 5.1.2 Procedure

Participants completed the VICE, then other questionnaires in three randomized blocks, and then demographics online. Items from the abbreviated Infrequency and Virtue scales of the Elemental Psychopathy Assessment were evenly interspersed throughout the questionnaires to assess data quality. We ensured our sample exceeded recommended thresholds for conducting CFAs on a 30‐item measure (i.e., 300 participants; Tinsley and Tinsley 1987).

#### 5.1.3 Measures

##### 5.1.3.1 VICE

Participants rated agreement with 30 items from 1 (*strongly disagree*) to 7 (*strongly agree*), assessing schadenfreude, gluckschmerz, and affective sadism (Table 3).

**TABLE 3:** Correlations between the Various Indices of Counter‐Empathy (VICE) and variables of interest.

	Various Indices of Counter‐Empathy (VICE)			
	Total	Schadenfreude	Gluckschmerz	Affective Sadism
Schadenfreude				
CSS Schadenfreude	0.71	0.64	0.56	0.46
SS Benign Schadenfreude	0.41	0.61	0.09	0.21
SS Malicious Schadenfreude	0.68	0.60	0.49	0.56
Counter‐Empathy				
ACME Affective Dissonance	0.74	0.63	0.53	0.63
Empathy				
ACME Cognitive Empathy	−0.20	−0.12	−0.18	−0.23
ACME Affective Resonance	−0.52	−0.47	−0.32	−0.49
IRI Empathic Concern	−0.43	−0.42	−0.26	−0.36
IRI Perspective Taking	−0.26	−0.22	−0.18	−0.24
Tall Poppy Syndrome				
TPS Favor Reward	−0.28	−0.30	−0.23	−0.09
TPS Favor Fall	0.50	0.42	0.44	0.31
Envy				
BeMaS Benign Envy	0.10	0.10	0.11	0.01
BeMaS Malicious Envy	0.62	0.38	0.66	0.47
Sadism				
SSIS Sadism	0.57	0.50	0.41	0.45
VAST Direct Sadism	0.61	0.55	0.37	0.58
VAST Vicarious Sadism	0.43	0.55	0.10	0.35
Aggression				
BAQ Anger	0.39	0.29	0.33	0.33
BAQ Hostility	0.40	0.29	0.41	0.25
BAQ Physical Aggression	0.29	0.36	0.12	0.19
BAQ Verbal Aggression	0.25	0.27	0.10	0.22
Narcissism				
FFNI Agentic Extraversion	0.07	0.02	0.06	0.13
FFNI Antagonism	0.59	0.51	0.41	0.54
FFNI Neurotic Narcissism	0.11	0.03	0.25	−0.05
Psychopathy				
EPA Antagonism	0.53	0.46	0.40	0.42
EPA Disinhibition	0.39	0.36	0.27	0.31
EPA Emotional Stability	−0.18	−0.14	−0.25	0.02
Machiavellianism				
FFMI Agency	−0.22	−0.14	−0.30	−0.06
FFMI Antagonism	0.49	0.46	0.33	0.37
FFMI Planfulness	−0.27	−0.22	−0.22	−0.20

*Note:* All correlations above |0.10| are significant at *p* ≤ 0.05 except VICE‐Schadenfreude with BeMaS Benign Envy and Gluckschmerz with BAQ Verbal Aggression.

##### 5.1.3.2 Chronic Schadenfreude Scale (CSS)

Participants rated agreement with 6 items (*α* = 0.84) on a scale from 1 (*strongly disagree*) to 7 (*strongly agree*) (Krizan and Johar 2012).

##### 5.1.3.3 Schadenfreude Scale (SS)

Participants rated agreement with 12 items (*α* = 0.84) from 1 (*strongly disagree*) to 9 (*strongly agree*) to assess benign schadenfreude, where little harm occurred (*α* = 0.83) and malicious schadenfreude, where noticeable harm occurred (*α* = 0.83) (Crysel and Webster 2018).

##### 5.1.3.4 Affective and Cognitive Measure of Empathy (ACME)

Participants rated agreement with 32 items (*α* = 0.90) from 1 (*strongly disagree*) to 5 (*strongly agree*) to assess cognitive empathy (*α* = 0.91), affective resonance (*α* = 0.86), and affective dissonance (*α* = 0.88; Vachon and Lynam 2016).

##### 5.1.3.5 Tall Poppy Scale

Participants rated agreement with 20 items (*α* = 0.90) from 1 (*I disagree a lot*) to 6 (*I agree a lot*). The Tall Poppy Scale (TPS) measures the preference for high achievers to fail (favor fall; *α* = 0.88) and succeed (favor reward; *α* = 0.85; Feather 1989).

##### 5.1.3.6 Benign and Malicious Envy Scale (BeMaS)

Participants rated agreement with 10 items (*α* = 0.83) from 1 (*strongly disagree*) to 6 (*strongly agree*): benign envy, wanting advantages of superior others while regarding them positively (*α* = 0.89), and malicious envy, hostility towards superior others and wanting to cut them down (*α* = 0.83; Lange and Crusius 2015).

##### 5.1.3.7 Interpersonal Reactivity Index (IRI)

Participants rated agreement with 28 items (*α* = 0.90) from 1 (*strongly disagree*) to 5 (*strongly agree*) assessing four empathic processes: perspective taking (*α* = 0.80), fantasy (*α* = 0.81), empathic concern (*α* = 0.89), and personal distress (*α* = 0.85; Davis 1980). We combined perspective taking and empathic concern to calculate total empathy scores (e.g., Wang et al. 2020).

##### 5.1.3.8 Short Sadistic Impulses Scale (SSIS)

Participants rated agreement with 10 items (*α* = 0.82) assessing sadism from 1 (*strongly disagree*) to 5 (*strongly agree*) (O'Meara et al. 2011).

##### 5.1.3.9 Varieties of Sadistic Tendencies (VAST)

Participants rated agreement with 16 items (*α* = 0.83) from 1 (*strongly disagree*) to 7 (*strongly agree*) assessing direct sadism (*α* = 0.73) and vicarious sadism (*α* = 0.81; Paulhus and Jones 2015).

##### 5.1.3.10 Brief Aggression Questionnaire (BAQ)

Participants rated how much they exhibit four indicators of aggression (*α* = 0.82) from 1 (*extremely uncharacteristic of* me) to 5 (*extremely characteristic of me*): anger (*α* = 0.78), hostility (*α* = 0.72), physical aggression (*α* = 0.88), and verbal aggression (*α* = 0.52; Webster et al. 2014).

##### 5.1.3.11 Five Factor Narcissism Inventory—Super Short Form (FFNI)

Participants rated agreement with 15 items (*α* = 0.67) from 1 (*strongly disagree*) to 5 (*strongly agree*) assessing agentic extraversion (*α* = 0.62), antagonism (*α* = 0.73), and neurotic narcissism (*α* = 0.85; Packer West et al. 2021).

##### 5.1.3.12 Five Factor Machiavellianism Inventory—Super Short Form (FFMI)

Participants rated agreement with 15 items (*α* = 0.62) from 1 (*strongly disa*gree) to 5 (*strongly agree*) assessing agency (*α* = 0.79), antagonism (*α* = 0.64), and planfulness (*α* = 0.81; Du et al. 2021).

##### 5.1.3.13 Elemental Psychopathy Assessment—Super Short Form (EPA)

Participants rated agreement with 18 items (*α* = 0.69) from 1 (*strongly disagree*) to 5 (*strongly agree*): antagonism (*α* = 0.69), disinhibition (*α* = 0.75), and emotional stability (*α* = 0.76; Collison et al. 2016).

##### 5.1.3.14 Rosenberg Self‐Esteem Scale

Participants rated agreement with 10 items capturing self‐esteem (*α* = 0.94) on a scale from 1 (*strongly disagree*) to 7 (*strongly agree*; Rosenberg 1965).

##### 5.1.3.15 Infrequency and Virtue Scales

Participants completed Infrequency and Virtue scales as in Study 1a and Study 1b.

### 5.2 Results and Discussion

Table 3 presents correlations between the VICE and other measures. VICE‐total and VICE‐schadenfreude correlate highly with other measures of schadenfreude. VICE‐schadenfreude may also differentiate schadenfreude from gluckschmerz more clearly than other measures: VICE‐schadenfreude correlates less with gluckschmerz (*r* = 0.38) than does CSS schadenfreude (*r* = 0.56; *z* = −4.93, *p* < 0.001) and SS malicious schadenfreude (*r* = 0.49; *z* = −2.77, *p* = 0.006). VICE‐total also correlates highly with affective dissonance, another overall measure of counter‐empathy. VICE‐total correlates negatively with cognitive (*r*'s from −0.20 to −0.26) and affective empathy (*r*'s from −0.43 to −0.52), though not so highly as to suggest redundancy.

VICE subscales also relate to counter‐empathic constructs as expected and without excessive overlap. Vicarious sadism, which is conceptually similar to schadenfreude, correlates more strongly with VICE‐schadenfreude than with VICE‐affective sadism. VICE‐gluckschmerz relates weakly to benign envy but strongly to malicious envy, suggesting a close conceptual relationship. Affective sadism correlates strongly with everyday sadism (SSIS *r* = 0.57; VAST‐direct sadism *r* = 0.61), which is expected given their shared focus on cruel behavior. Notably, these sadism measures correlate highly with VICE‐schadenfreude as well, suggesting significant conceptual or item‐content overlap between these measures.

VICE scores also relate to measures of socially aversive behaviors and personalities. VICE‐total correlates strongly with antagonism in all Dark Triad measures (*r*'s from 0.49 to 0.59), which has been posited to be the “core” of dark personalities (Vize et al. 2020). The VICE correlates less strongly with other Dark Triad subscales (*r*'s from −0.27 to 0.39; see Table S4). The VICE correlated positively with all aspects of aggression.

#### 5.2.1 Incremental Validity Analysis

**FIGURE 1:**
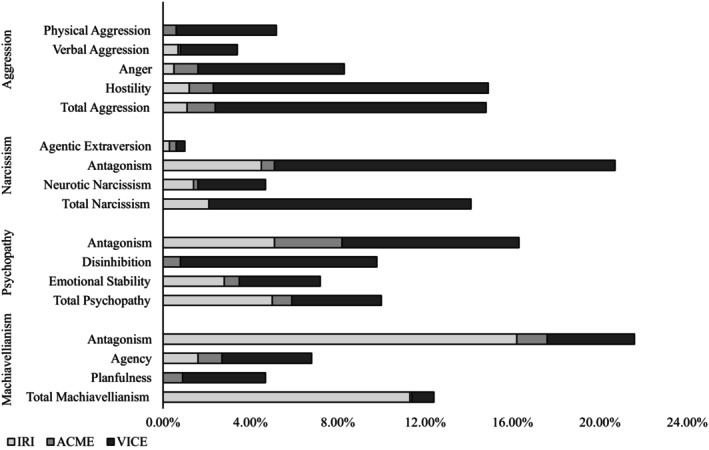
Incremental prediction of social aversive personalities and behavior by empathy and counter‐empathy: Δ*R* ^2^ when entered in the last step of a hierarchical regression.

To test whether total counter‐empathy incrementally predicts socially aversive behaviors and personalities beyond total empathy, we conducted sets of hierarchical regression analyses, each with one particular measure entered at the last step; the *R* ^2^‐change then quantified the unique variance of the outcome shared with that variable. For these analyses, ACME scores were calculated using the cognitive empathy and affective resonance subscales only (*α* = 0.90), to isolate subscales assessing empathy rather than counter‐empathy. Figure 1 presents the incremental contributions of the IRI, ACME, and VICE to each outcome, with indicated sections of each bar representing that measure's unique variance. The VICE had the largest unique contributions to predicting aggression, narcissism, the antagonism, disinhibition, and emotional stability subscales of the EPA, and the agency and planfulness subscales of the FFMI. These results demonstrate the incremental validity of the VICE and the relevance of counter‐empathy beyond empathy in predicting these outcomes. Notably, even in cases where the VICE was not the largest incremental predictor, its unique contribution was significant.

#### 5.2.2 Confirmatory Factor Analysis

Through confirmatory factor analyses (CFAs) we tested a three‐factor model—with schadenfreude, gluckschmerz, and affective sadism items loading on distinct, correlated latent variables—using polychoric correlation matrices with weighted least square means and adjusted variance (WLSMV) estimators (to account for the non‐continuous nature of the Likert scales). This model fit well (Hu and Bentler 1999): *χ* ^2^(402) =1410.61, *p* < 0.001, CFI = 0.97, TLI = 0.96, RMSEA = 0.08, and better ($\chi_{\mathrm{dif}}^{2}$(3) = 335.99, *p* < 0.001) than a one‐factor model (CFI = 0.88, TLI =0.88, RMSEA = 0.15). We additionally tested a bifactor model, in which a general counter‐empathy factor was added alongside the three specific factors. This bifactor model also fit well: χ^2^(375) = 1136.59, *p* < 0.001, CFI = 0.97, TLI = 0.97, RMSEA = 0.07, and better than the three‐factor model ($\chi_{\mathrm{dif}}^{2}$(27) = 217.75, *p* < 0.001). These results support the three‐factor model specified by the 3DCE, with a hierarchical structure supporting a general disposition of counter‐empathy with more specific facets of schadenfreude, gluckschmerz and affective sadism.

#### 5.2.3 Hierarchical Structure of Counter‐Empathy

No prior research has examined the emergent structure of dispositional counter‐empathy and how lower‐order counter‐empathic constructs relate to a broader disposition. To understand the structure of counter‐empathy and provide a reference to organize work across substantive areas of study, we conducted a “bass‐ackward” factor analysis (Goldberg 2006). This approach employs EFA analyses to examine factors extracted from scale items derived from a relatively comprehensive sample of scales in a particular substantive area; in this case, scales that assess counter‐empathic constructs. This technique maps the hierarchical structure of broader, high‐level factors relative to lower‐order, more specific factors, examining how these factors relate across different levels of abstraction. Specifically, a series of EFAs are conducted where additional factors are progressively extracted until the factors are no longer interpretable. Factor solutions at each stage can then be compared to generate a hierarchical model that describes how dispositional counter‐empathy breaks apart into lower‐order constructs. This process allows us to map the structure of counter‐empathy as it exists in the literature and compare it to the 3DCE.

We included the ACME‐Affective Dissonance subscale, BeMaS, CSS, SS, SSIS, TPS, VAST, and VICE. Though everyday sadism is not inherently counter‐empathic, we include measures of it to test its distinctness from affective sadism (note, a parallel analysis that excludes everyday sadism measures produced highly consistent results for the remaining measures). Items that loaded less than |0.30| on the single‐factor solution were first removed (Crowe et al. 2018). This removed all items from the benign envy subscale of the BeMaS and the favor reward subscale of the TPS. Subsequently, six items were removed from SS benign schadenfreude and the VAST. To prevent the emergence of bloated specific factors, highly overlapping items were removed, using a cutoff of *r* > 0.80. One item from each pair of overlapping items was removed in stepwise fashion until no overlapping items remained. This removed three items from the VICE. All factor solutions were extracted using PAF and with a Promax Rotation.

**FIGURE 2:**
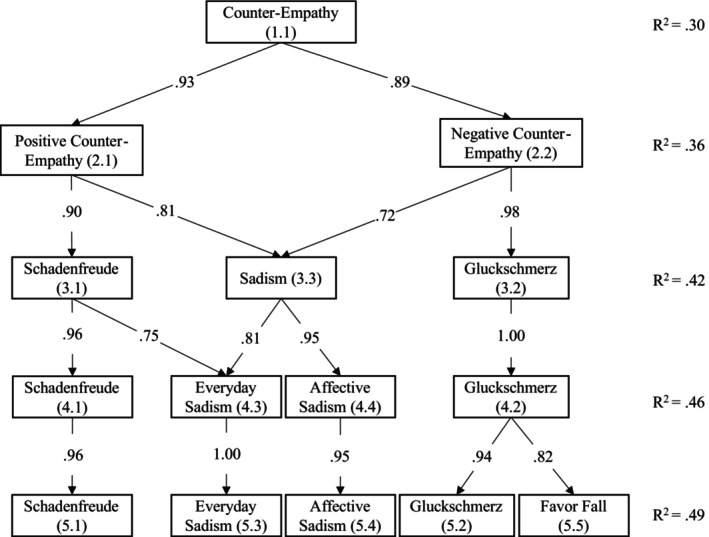
Hierarchical structure of counter‐empathy. Only correlations going from one level of the hierarchy to the next are depicted. Correlations less than 0.70 are not displayed.

The first 10 eigenvalues ranged from 27.87 to 1.49 (See Figure 2). A parallel analysis suggested retaining nine factors, whereas a MAP analysis suggested eight factors. After examining the item content and factor loadings of each solution, a five‐factor solution was most interpretable and parsimonious (see Tables S5 and S6 for all factor loadings and intercorrelations between factors). To help interpret the content of each factor, we examined correlations between factor scores and complete scales (Table 4). For more complex factor solutions, the sixth factor consisted of three VAST‐vicarious sadism items and the seventh factor consisted of strongly worded items from CSS‐schadenfreude, SS‐malicious schadenfreude, BeMaS‐malicious envy, and ACME‐affective dissonance.

**TABLE 4:** Correlations between fifth‐level Bass‐Ackward factors and included measures.

Variable	F5.1	F5.2	F5.3	F5.4	F5.5
VICE Schadenfreude	**0.93**	0.47	0.59	0.45	0.47
VICE Gluckschmerz	0.38	**0.93**	0.44	0.47	0.45
VICE Affective Sadism	0.52	0.61	0.56	**0.93**	0.33
VICE Total Counter‐Empathy	0.79	0.83	0.66	0.70	0.53
ACME Affective Dissonance	0.74	0.65	0.89	0.59	0.50
BeMaS Malicious Envy	0.45	0.83	0.56	0.34	0.68
CSS Schadenfreude	0.75	0.75	0.67	0.30	0.69
SS Benign Schadenfreude	0.71	0.16	0.34	0.19	0.30
SS Malicious Schadenfreude	0.73	0.65	0.66	0.50	0.53
SS Total Schadenfreude	0.85	0.42	0.55	0.37	0.46
TPS Favor Fall	0.51	0.53	0.42	0.24	**0.97**
SSIS Sadism	0.56	0.50	**0.90**	0.40	0.42
VAST Direct Sadism	0.62	0.46	0.81	0.59	0.46
VAST Vicarious Sadism	0.63	0.10	0.50	0.46	0.34
VAST Total Everyday Sadism	0.73	0.29	0.73	0.59	0.45

*Note:* All correlations equal to or greater than |0.10| are significant at *p* ≤ 0.05. The largest facet‐level correlation for each factor is in bold.

At the first level, the factor (F1.1, 30% of total variance) was labeled Counter‐Empathy, reflecting the fundamental aspect shared by all items, with highest loading items reflecting schadenfreude, gluckschmerz, and sadism. At the second level (36% of total variance), Counter‐Empathy broke into Positive Counter‐Empathy (F2.1), reflecting the tendency to feel positively in response to witnessing or causing another's misfortune (with schadenfreude and vicarious sadism items loading most highly), and Negative Counter‐Empathy (F2.2), the tendency to feel negatively in response to another's good fortune (with gluckschmerz and malicious envy items loading most highly). *At the* third level (42% of total variance), Positive Counter‐Empathy was mostly retained, being labeled Schadenfreude (F3.1), and Negative Counter‐Empathy was mostly retained, being labeled Gluckschmerz (F3.2). However, a combination of second level factors emerged, labeled Sadism (F3.3), with affective sadism and everyday sadism items loading most highly. At the fourth level (46% of total variance), Schadenfreude and Gluckschmerz were mostly retained, though Sadism split into factors labeled Everyday Sadism (F4.3) and Affective Sadism (F4.4) with the highest loading items reflecting these constructs. At the fifth level (49% of total variance), all higher‐order factors were retained, with a new factor emerging labeled Favor Fall (F5.5), reflecting a desire for individuals of high status to lose their social standing.

These results are notable for several reasons. As the factor structure becomes more differentiated (levels 4 and 5), all VICE items load distinctly on their intended factors (e.g., all VICE‐schadenfreude items load on the schadenfreude factor). Other measures of schadenfreude include items that load on gluckschmerz rather than schadenfreude. The Affective Dissonance subscale of the ACME may reasonably assess overall counter‐empathy (with items that load on schadenfreude, gluckschmerz and affective sadism) but many of its items primarily load on everyday sadism. In fact, ACME‐affective dissonance loaded more strongly on everyday sadism than did the VAST. Notably, everyday sadism separated into its own factor, with items from SSIS‐Sadism and VAST‐direct sadism loading strongly on it. Items from VAST‐vicarious sadism, however, loaded on schadenfreude. Overall, the results support the 3DCE and suggest the VICE assesses its dimensions cleanly. The results at levels 4 and 5 also suggest that Favor Fall is most closely associated with gluckschmerz but may have enough unique content that it can be reasonably studied as a distinct phenomenon (likely due to its focus on others' status).

Notably, malicious envy correlated highly with gluckschmerz and did not separate from it, indicating substantial empirical overlap. One interpretation is that malicious envy is a specific manifestation of gluckschmerz, which is a more general counter‐empathic reaction to others' good fortune. Alternatively, some malicious envy items may capture gluckschmerz, rather than a desire to gain another person's superior achievement or outcome (e.g., “Seeing other people's achievements makes me resent them”).

## 6 Study 3a and Study 3b

Studies 3a and 3b test whether counter‐empathy, as assessed by the VICE, predicts specific responses to others' outcomes. In Study 3a, participants read about others' good fortunes or misfortunes and rated how they would feel seeing them. Higher dispositional schadenfreude should predict more positive affect in response to misfortunes, whereas higher gluckschmerz should predict negative affect in response to good fortunes. We also test these effects controlling for benign and malicious envy and empathy to test their robustness. In Study 3b, participants read and reacted to vignettes of others' misfortunes that have successfully elicited schadenfreude in past research. We conducted Study 3b because VICE‐schadenfreude did not clearly predict positive reactions to misfortunes in Study 3a, and we wanted to test if this was due to the specific vignettes used in Study 3a. We also compare how well VICE‐schadenfreude predicts positive affect in response to misfortunes compared to other dispositional schadenfreude measures.

In Study 3a, we also further test the distinctness of affective sadism and everyday sadism. Affective sadism is meant to specifically entail a response to others' emotions. Participants read about targets inflicting harm on others who were either not clearly displaying emotion (i.e., general harm) or who were experiencing strong emotions (i.e., emotional harm). Those higher in affective sadism should report more positive affect and that they are more likely to act similarly in response to emotional harm compared to general harm. We also expect these relations to hold when controlling for everyday sadism. In contrast, everyday sadism should predict positive reactions and being likely to act similarly in both emotional and general harm scenarios.

### 6.1 Method

#### 6.1.1 Participants

##### 6.1.1.1 Study 3a

Canadian undergraduates (*N* = 454) participated for partial course credit. We ensured samples exceeded 250 participants, the sample size at which correlations and regression coefficients are expected to stabilize (Schönbrodt and Perugini 2013). However, we deliberately oversampled in anticipation of data exclusions. Using preregistered criteria, participants were excluded for reporting their data should not be used (*n* = 12), inattentive responding (*n* = 19), responding overly quickly (< 650 s; *n* = 22), or overly slowly (> 3 h; *n* = 23). We did not use one preregistered exclusion criterion that involved responding accurately to a written passage; few participants completed it correctly, which we attributed to the subtlety of the check rather than poor data quality. The final sample consisted of 378 participants (12.7% male, 85.7% female, 1.3% non‐binary, and 0.3% other) ranging in age from 17 to 63 (*M* = 19.99, SD = 3.34) and identifying their ethnicity as: White (63.8%), South Asian (14.0%), East Asian (4.0%), Middle Eastern (3.4%), Latin, Central, or South American (2.6%), African (2.6%), Caribbean (2.4%), Indigenous (0.5%), and other (6.6%). The preregistration, data, analysis code, and complete surveys are at: https://osf.io/x6wy2/

##### 6.1.1.2 Study 3b

Canadian adults (*N* = 352) recruited through CloudResearch Connect received 2.50 USD. Participants were adults instead of undergrads, deviating from our preregistration. We conducted an initial version of 3b with undergraduates but administered the VICE with subscale items interspersed rather than blocked, which greatly inflated their interrelations. Results were otherwise identical to those reported here (see Supporting Information). We replicated the study with blocked items but recruited participants through CloudResearch to expedite data collection. Using preregistered criteria, participants were excluded for inattentive responding (*n* = 16) and responding overly slowly (> 45 min; *n* = 5). The final sample consisted of 331 participants (54.4% male, 45.6% female) ranging in age from 18 to 71 (*M* = 33.32, SD = 11.15) and identifying their ethnicity as: White (54.1%), East Asian (21.1%), South Asian (10.0%), African (3.0%), Caribbean (3.0%), Middle Eastern (2.7%), Indigenous (1.2%), Latin, Central, or South American (0.9%), and other (3.9%). The preregistration, data, analysis code, and complete surveys are at: https://osf.io/dbwa2/

#### 6.1.2 Procedure

In both studies, participants completed personality measures and responded to vignettes online, in counterbalanced order. At the end of the study, they reported demographics. In Study 3b, the vignettes were presented as diary entries recorded by participants in a previous study so that participants would view them as events that actually occurred to someone else.

#### 6.1.3 Vignettes

In Study 3a, participants read two sets of vignettes. In the first set, four vignettes depict targets experiencing good fortune (e.g., receiving a scholarship) and four other vignettes depict misfortune (e.g., having a comedy routine fall flat). We also manipulated the apparent morality of targets, but this did not moderate any results (see Supporting Information). In Study 3b, participants read eight vignettes depicting misfortune (Greenier 2021). After each vignette, participants rated how “Pleased,” “Happy,” and “Joyful” (Kinrade et al. 2022) the event would make them and, in Study 3a, how “Irritated,” “Annoyed,” and “Upset,” or, in Study 3b, how “Sympathetic,” “Concerned,” and “Empathetic,” (Batson et al. 1981) from 1 (*not at all*) to 5 (*extremely*). These items were averaged as indexes of positive affect (misfortune *α* = 0.82; good fortune *α* = 0.86; Study 3b *α* = 0.90), negative affect (misfortune *α* = 0.84; good fortune *α* = 0.87), or empathic affect (Study 3b; *α* = 0.94).

The second set in Study 3a depicted targets inflicting harm on others, with participants imagining themselves as the person inflicting harm. Four vignettes depict “affective harm,” in response to others' positive or negative emotion such as undermining happiness or amplifying distress (e.g., belittling an achievement someone expressed pride in), and four depict “general harm,” which was not in response to others' emotion (e.g., mocking someone's appearance; Kinrade et al. 2022). After each vignette, participants indicated how they would feel if they were the person inflicting harm, using the same items as above (positive affect: affective harm *α* = 0.95; general harm *α* = 0.94; negative affect: affective harm *α* = 0.95; general harm *α* = 0.94). Participants also rated how likely they would be to inflict harm in the way described on 3 items (e.g., “How likely is it that you would behave in the way described in the scenario?”) from 1 (*not at all*) to 5 (*extremely*) as an index of willingness to harm (affective harm *α* = 0.92; general harm *α* = 0.92).

#### 6.1.4 Personality Measures

##### 6.1.4.1 Various Indices of Counter‐Empathy (VICE)

In both studies, participants completed the VICE as in Study 2. Table 2 presents the reliabilities of the VICE in these studies.

##### 6.1.4.2 Rosenberg Self‐Esteem Scale (RSES)

In both studies, participants completed the RSES (Study 3a: *α* = 0.92; Study 3b: *α* = 0.94) as in Study 2 to assess data quality.

##### 6.1.4.3 Demographic Questionnaire

In Study 3a, participants completed a 6‐item questionnaire assessing their age, gender, ethnicity, academic major, year of study, and sexuality. In Study 3b, participants completed a 3‐item questionnaire assessing their age, gender, and ethnicity.

##### 6.1.4.4 Comprehensive Assessment of Sadistic Tendencies (CAST)

In Study 3a, participants rated agreement with 12 items (*α* = 0.82) from 1 (*strongly disagree*) to 5 (*strongly agree*) assessing direct physical sadism (*α* = 0.80), direct verbal sadism (*α* = 0.78), and vicarious sadism (*α* = 0.83; Buckles 2023).

##### 6.1.4.5 Empathy Quotient—Short (EQ)

In Study 3a, participants rated agreement with 22 items (*α* = 0.71) from 1 (*definitely disagree*) to 4 (*definitely agree*) (Wakabayashi et al. 2006).

##### 6.1.4.6 Benign and Malicious Envy Scale

In Study 3a, participants completed the Benign and Malicious Envy Scale (BeMaS) as in Study 2 (total BeMaS *α* = 0.84; benign envy *α* = 0.90; malicious envy *α* = 0.87).

##### 6.1.4.7 Chronic Schadenfreude Scale

In Study 3b, participants completed the Chronic Schadenfreude Scale (CSS; *α* = 0.85) as in Study 2.

##### 6.1.4.8 Schadenfreude Scale

In Study 3b, participants completed the Schadenfreude Scale (SS) as in Study 2 (total SS *α* = 0.82; benign schadenfreude *α* = 0.78; malicious schadenfreude *α* = 0.75).

##### 6.1.4.9 Varieties of Sadistic Tendencies

In Study 3b, participants completed the Varieties of Sadistic Tendencies (VAST) as in Study 2 (total VAST *α* = 0.80; direct sadism *α* = 0.79; vicarious sadism *α* = 0.71).

##### 6.1.4.10 Questionnaire of Cognitive and Affective Empathy (QCAE)

In Study 3b, participants rated agreement with 31 items from 1 (*strongly disagree*) to 4 (*strongly agree*) assessing two forms of empathy (*α* = 0.91): cognitive empathy (*α* = 0.93) and affective empathy (*α* = 0.78).

### 6.2 Results and Discussion

Correlations between the VICE and other personality variables appear in Table 5. In Study 3a, the VICE total was unrelated to empathy, but in Study 3b, VICE total related negatively to cognitive (*r* = −0.20) and affective empathy (*r* = −0.18). Correlations with everyday sadism and envy range from 0.08 to 0.67, with the highest being between VICE affective sadism and CAST direct physical sadism, and VICE gluckschmerz and malicious envy. The VICE and its subscales correlated with all measures of schadenfreude in Study 3b.

**TABLE 5:** Correlations between the Various Indices of Counter‐Empathy and Personality Variables.

Variable	Various Indices of Counter‐Empathy (VICE)			
	Total	Schadenfreude	Gluckschmerz	Affective sadism
*Study 3a*				
Envy				
BeMaS Benign Envy	0.18	0.13	0.17	0.11
BeMaS Malicious Envy	0.66	0.39	0.66	0.49
Sadism				
CAST Direct Physical Sadism	0.51	0.29	0.35	0.67
CAST Direct Verbal Sadism	0.42	0.42	0.23	0.35
CAST Vicarious Sadism	0.43	0.45	0.20	0.39
Empathy				
EQ Empathy	−0.06	−0.02	−0.04	−0.11
*Study 3b*				
Schadenfreude				
CSS Schadenfreude	0.77	0.56	0.66	0.55
SS Benign Schadenfreude	0.46	0.66	0.16	0.24
SS Malicious Schadenfreude	0.69	0.52	0.57	0.53
Sadism				
VAST Vicarious Sadism	0.37	0.53	0.08	0.27
VAST Direct Sadism	0.61	0.44	0.40	0.66
Empathy				
QCAE Cognitive Empathy	−0.20	−0.11	−0.22	−0.12
QCAE Affective Empathy	−0.18	−0.19	−0.06	−0.21

*Note:* All correlations above |0.10| are significant at *p* ≤ 0.05 except VICE Schadenfreude with QCAE Cognitive Empathy.

#### 6.2.1 Fortune Vignettes

Contrary to expectations, in Study 3a, VICE‐schadenfreude did not relate to positive affect in response to others' misfortunes, although VICE‐total, gluckschmerz and affective sadism did (Table 6). The lack of relation for schadenfreude may be due, in part, to the specific scenarios used in this study. In Study 3b, we adapted eight misfortune vignettes that have elicited feelings of schadenfreude in past research (Greenier 2021); this also allowed us to sample a wider variety of misfortunes. VICE‐schadenfreude predicted positive affect in response to these vignettes, as did the other VICE subscales. The relation with VICE‐schadenfreude remained significant when controlling cognitive empathy, *β* = 0.09, *t*(328) = 7.27, *p* < 0.001, and affective empathy, *β* = 0.09, *t*(328) = 7.01, *p* < 0.001. This relationship was also similar to that observed for other schadenfreude measures (CSS *r* = 0.41; SS‐Total *r* = 0.36; SS‐Benign *r* = 0.21; SS‐Malicious *r* = 0.43; see Table S7). Overall, VICE‐schadenfreude predicts positive reactions to others' misfortune. Notably, VICE‐total, gluckschmerz and affective sadism also predict such reactions, supporting that these dimensions of counter‐empathy form a coherent disposition.

**TABLE 6:** Correlations between the Various Indices of Counter‐Empathy and vignette reactions.

Variable	Various indices of counter‐empathy (VICE)			
	Total	Schadenfreude	Gluckschmerz	Affective sadism
Good fortune vignettes				
Positive Affect	0.03	−0.01	0.02	0.08
Negative Affect	0.13*	−0.02	0.21**	0.12*
Misfortune vignettes				
Study 3a				
Positive Affect	0.18**	0.08	0.18**	0.16*
Negative Affect	0.07	−0.03	0.08	0.16*
Study 3b				
Positive Affect	0.55**	0.38*	0.41**	0.55*
Empathic Affect	−0.16	−0.21	−0.08	−0.07

Emotional harm vignettes				
Positive Affect	0.18**	0.13*	0.10*	0.22**
Negative Affect	−0.06	−0.13*	0.01	−0.02
Willingness to Harm	0.39**	0.23**	0.30**	0.46**
General harm vignettes				
Positive Affect	0.24**	0.20**	0.14*	0.24**
Negative Affect	−0.05	−0.13*	0.00	−0.03
Willingness to Harm	0.40**	0.28**	0.27**	0.45**

* *p* ≤ 0.05.

** *p* ≤ 0.001.

As expected, in Study 3a, VICE‐gluckschmerz relates to negative affect in response to good fortunes, as do VICE‐total and affective sadism. The relation with gluckschmerz remained significant when controlling benign envy, *β* = 0.15, *t*(375) = 3.81, *p* < 0.001, and empathy, *β* = 0.16, *t*(375) = 4.22, *p* < 0.001, but not malicious envy, *β* = 0.08, *t*(375) = 1.63, *p* = 0.104. As noted, this may be due to the malicious envy measure including items that assess gluckschmerz. The good fortune scenarios, moreover, describe desirable outcomes (e.g., winning the lottery) that may evoke envy. Controlling malicious envy may attenuate the relation with gluckschmerz, because malicious envy is a narrower construct than gluckschmerz (Paunonen et al. 2003). In general, VICE‐gluckschmerz predicts specific negative reactions to others' good fortune.

#### 6.2.2 Harm Vignettes

Participants higher in affective sadism reported more positive affect in response to the emotional harm vignettes and reported being more willing to inflict similar harm (Table 6). Those high in everyday sadism (CAST‐total) also reported positive affect (*r* = 0.13) and willingness to harm (*r* = 0.37) in response to the emotional harm vignettes. Affective sadism relates more strongly than everyday sadism to willingness to harm (*z* = 2.03, *p* = 0.042), but not positive affect (*z* = 1.88, *p* = 0.060). In addition, the relation of affective sadism to positive affect remains significant when controlling everyday sadism, *β* = 0.17, *t*(375) = 3.49, *p* < 0.001, and empathy, *β* = 0.17, *t*(375) = 4.32, *p* < 0.001. In contrast, with affective sadism controlled, everyday sadism does not predict positive affect, *β* = 0.01, *t*(375) = 0.15, *p* = 0.88. Affective sadism also predicts willingness to inflict harm with everyday sadism, *β* = 0.20, *t*(375) = 6.69, *p* < 0.001, and empathy, *β* = 0.24, *t*(375) = 9.81, *p* < 0.001 controlled. For responses to general harm, affective sadism and everyday sadism both predict positive affect (*r* = 0.24 and *r* = 0.22, respectively) and willingness to harm (*r* = 0.41 and *r* = 0.44) about equally. Notably, VICE‐schadenfreude does predict positive affect in response to both emotional and general harm vignettes.

These results partially support our predictions: Everyday sadism and affective sadism predict positive affect in response to both general and emotional harm and greater willingness to inflict both kinds of harm. This suggests the two dispositions are closely related; those high in one form of sadism are likely high in the other form (*r* = 0.56). However, affective sadism relates more strongly than everyday sadism to the willingness to inflict emotional harm. In addition, affective sadism relates to positive affect and the willingness to inflict emotional harm with everyday sadism controlled. Overall, these results support a unique relationship between affective sadism and the willingness to inflict emotional harm.

## 7 General Discussion

Dispositional counter‐empathy has been studied—directly or indirectly—across multiple lines of research involving different constructs such as schadenfreude, envy, gluckschmerz, Tall Poppy Syndrome, and affective dissonance. Its study is also closely linked to the study of everyday sadism (Parton and Chester 2025). To help bring greater conceptual clarity to the study of counter‐empathy, we introduce the 3DCE, which proposes schadenfreude, gluckschmerz, and affective sadism as cardinal dimensions of dispositional counter‐empathy. Our studies support this model while developing a multidimensional measure of counter‐empathy, the VICE.

EFAs and CFAs, on items developed for the VICE to assess the 3DCE, support its three‐factor structure. A bifactor model fit the VICE well, supporting a hierarchical structure in which dispositional schadenfreude, gluckschmerz, and affective sadism represent specific facets of a general counter‐empathy disposition. This hierarchical structure is further supported by a “bass‐ackward” factor analysis, which also supports the distinctness of affective sadism and everyday sadism. The VICE and its subscales correlate as expected with other measures of counter‐empathy (e.g., affective dissonance; schadenfreude) and theoretically related constructs (e.g., envy, everyday sadism). The VICE is also distinct from empathy and predicts substantial unique variance in aggression and Dark Triad traits. Lastly, VICE‐schadenfreude and gluckschmerz predict specific counter‐empathic responses to others' misfortunes, and VICE‐affective sadism predicts greater willingness to inflict harm on emotional others (more so than others in general). Overall, these findings support the 3DCE and the validity of the VICE for assessing it.

### 7.1 3DCE as a Framework for Counter‐Empathy Research

A key aim of the current research was to develop a structural framework that can help conceptually clarify and integrate separate research programs on counter‐empathic constructs. Our bass‐ackward factor analysis, containing measures of major counter‐empathic constructs, revealed multiple distinguishable dimensions of counter‐empathy and provided empirical support for the 3DCE. These results can help organize and conceptually bridge distinct areas of research. For example, measures of both malicious envy and Tall Poppy Syndrome aligned with the gluckschmerz factor, suggesting they may reflect a common disposition. Research on one could therefore inform understanding of the other: the fact that those high in Tall Poppy Syndrome tend to be lower in self‐esteem (e.g., Feather 1989) may suggest that those high in malicious envy, or dispositional gluckschmerz, are similarly low in self‐esteem. Similarly, vicarious sadism appears to align more closely with schadenfreude, as both involve vicarious enjoyment of others' suffering, than with everyday sadism, which involves inflicting suffering. The 3DCE can therefore help organize and consolidate research on different counter‐empathic constructs. It could be used to situate additional constructs that may relate to dispositional counter‐empathy, such as contempt (Hornik et al. 2021), gloating (Leach et al. 2015), or resentment (Feather et al. 2013).

At the level of measurement, patterns of correlations of other measures with VICE subscales, as well as the results of the bass‐ackward factor analysis, suggest some measures of counter‐empathic constructs assess multiple 3DCE dimensions or everyday sadism. This can create ambiguity in how to best interpret them. Other measures of schadenfreude, for example, include items that appear to assess gluckschmerz (Krizan and Johar 2012). The affective dissonance subscale of the ACME includes items that appear to assess everyday sadism (Vachon and Lynam 2016). Although the Favor Fall subscale of the TPS loads most clearly on our gluckschmerz factor, the scale correlates highly with both VICE‐gluckschmerz and VICE‐schadenfreude, includes items that cross‐load on both, and items that may be an amalgam of both (e.g., “It's good to see very successful people fail occasionally”) because they suggest resenting others' success and enjoying their downfall. Based on the 3DCE, the VICE subscales offer more clearly delineated assessments of counter‐empathic dimensions, including the first measures of dispositional gluckschmerz and affective sadism.

### 7.2 Counter‐Empathy Is Not (Low) Empathy

Across studies, we administered four different measures of empathy alongside the VICE. Empathy related negatively to counter‐empathy in three studies, though not so highly as to be redundant, and was unrelated in another. The VICE accounts for substantial unique variance with socially aversive behavior and personalities with empathy controlled. We replicate the finding that counter‐empathy uniquely predicts aggression beyond empathy (Vachon and Lynam 2016). That earlier finding, however, was based on the incremental contribution of the ACME (including affective dissonance) to predicting aggression over other empathy measures. Our analyses suggest that ACME‐affective dissonance includes items that assess everyday sadism, raising the possibility that everyday sadism, rather than counter‐empathy, uniquely predicts aggression. Our incremental validity analyses demonstrate that counter‐empathy (the VICE) specifically predicts unique variance in aggression beyond empathy. Further supplemental analyses reveal both VICE‐schadenfreude (*β* = 0.19) and gluckschmerz (*β* = 0.11) uniquely predict aggression (BAQ‐total) with empathy controlled (Table S8). These findings provide evidence that the relation of counter‐empathy to aggression is not due to active counter‐empathy or sadism; passive counter‐empathy also predicts aggression beyond empathy.

These findings support the view that counter‐empathy is a better predictor of socially aversive behavior than is a lack of empathy (e.g., Hudson et al. 2022). Empathy has been argued to be a key predictor of the benevolent or malevolent nature of individual behavior (e.g., Baron‐Cohen 2011), where deficits in empathy contribute to socially aversive acts (e.g., Miller and Eisenberg 1988; Paulhus 2014). However, empathy is a weak predictor of socially aversive behavior such as aggression (Vachon et al. 2014). Our findings (and those of Vachon and Lynam 2016) provide preliminary evidence that counter‐empathy contributes to aggression and other undesirable social acts. Certainly, further research with behavioral measures is warranted; nonetheless, these findings may inform the development of interventions to reduce such behaviors through empathy development (Weisz and Zaki 2017). It may be most productive to supplement such efforts by developing interventions designed to decrease counter‐empathy.

Our results also suggest that counter‐empathy characterizes dark personalities. A lack of empathy or callousness has been described as a prominent aspect of the “core” of Dark Triad traits (Jones and Figueredo 2013; Paulhus 2014). We found the VICE correlates highly with the antagonism subscales of all Dark Triad measures—antagonism being another proposed core feature of the Dark Triad (Vize et al. 2020)—even with empathy controlled. Considerable debate continues over which attributes constitute the core of dark personalities (e.g., Jones and Figueredo 2013; Lynam and Miller 2019; Vize et al. 2020); our results, however, suggest that counter‐empathy may be a common feature of dark personalities. The empathic “deficits” that characterize these personalities may go beyond callousness or indifference to encompass counter‐empathy (Vachon and Lynam 2016).

### 7.3 Distinctiveness of 3DCE Dimensions

We include gluckschmerz in the 3DCE rather than envy, as it is conceptually broader. Though gluckschmerz is distinct from benign envy, it overlaps with malicious envy: The two correlate highly and fail to separate in the bass‐ackward factor analysis. VICE‐gluckschmerz predicts negative reactions to others' specific good fortunes, but this relation does not persist with malicious envy controlled. These results might not be entirely surprising. As noted, some malicious envy items seem to assess feeling negatively towards envied or superior others (e.g., “Seeing other people's achievements makes me resent them”) rather than envy per se. Although benign envy involves longing for another's advantage, malicious envy involves wanting their downfall, which is similar to gluckschmerz. Disentangling envy and gluckschmerz may be difficult because others' good fortunes may often elicit social comparison, making envy a more dominant response than gluckschmerz (Smith and van Dijk 2018). Future research should probe their distinctness further, testing, for example, whether dispositional gluckschmerz predicts negative reactions to forms of good fortune that malicious envy does not—such as positive outcomes that one does not want for oneself (e.g., an acquaintance praised as a guitar prodigy, even if one does not aspire to play guitar well). Regardless, our results suggest research on malicious envy is relevant to understanding dispositional gluckschmerz and vice versa.

There is also a strong, though more clearly distinct, relationship between affective sadism and everyday sadism. Smith and van Dijk (2018) theorized counter‐empathy includes “a hostile action tendency or hostile disposition” (p. 297). We conceptualize affective sadism as a narrow form of sadistic pleasure‐seeking and an active form of counter‐empathy. Affective and everyday sadism separated into distinct factors in the bass‐ackward factor analysis. Individuals high in affective sadism indicated a greater willingness to inflict specific emotional harms on others, more so than the willingness to inflict general harm. The relation between affective sadism and being willing to inflict emotional harm also persisted with everyday sadism controlled, but everyday sadism did not predict willingness to inflict emotional harm with affective sadism controlled. Our results suggest affective sadism is relatively specific to harming others who are emotional, whereas those high in everyday sadism are prone to inflict harm regardless of context. They also suggest that dispositional counter‐empathy includes an active dispositional tendency. Further work should examine whether and how this tendency may arise from passive counter‐empathy.

### 7.4 Limitations

These studies support the 3DCE as a structural model of dispositional counter‐empathy, as well as the validity of the VICE. Although our empirical analyses support the 3DCE, we initially identified its dimensions through theoretical, top‐down consideration of research and theory and developed items specifically to assess these dimensions. This means our EFAs and CFAs included only items that assess 3DCE dimensions. The bass‐ackward factor analysis incorporated items from other measures of counter‐empathic constructs, but this method also estimates counter‐empathy as a culmination of the specific items included in the analysis. If other—potentially overlooked—aspects of counter‐empathy were included, the resultant factor structure could change. The analysis is also limited by the number of items that represent each construct. For instance, malicious envy was only represented by five items, which may have limited its ability to emerge as a distinct factor. As more measures of counter‐empathy are developed, there should be further efforts to map the hierarchical structure of counter‐empathy.

A further limitation is reliance on self‐report measures, which can be susceptible to social desirability biases, especially given counter‐empathy is a relatively taboo topic (Krumpal 2013). However, correlations observed between the VICE, socially desirable traits (e.g., empathy) and undesirable traits (e.g., aggression) are consistent with past work. We also presented vignettes in Study 3b as events that had happened to prior participants, increasing confidence that affective responses to them were genuine. The measure of aggression also relates to behavioral aggression (Webster et al. 2014). Nevertheless, relations of dispositional counter‐empathy to important behavioral outcomes, as well as more naturalistic reactions to others' good fortunes and misfortunes, should be assessed in future studies. This work focused on how counter‐empathy related to aversive outcomes. Future work should examine other (especially positive) outcomes.

Our samples span both undergraduates and adults from Canada and the United States. Their ages, gender, and ethnic identities suggest our samples are diverse, but they included only participants from WEIRD (Western, educated, industrialized, rich, and democratic) cultures (Henrich et al. 2010). Our US samples were also sampled from an online crowdsourcing platform (CloudResearch Connect) and may thus have different personality characteristics than the general population (Colman et al. 2018). Our findings should not be assumed to generalize to non‐WEIRD populations or other populations outside those sampled for this study. Future research should test the 3DCE and the validity of the VICE in additional populations.

## 8 Conclusions

We introduce the 3DCE as a comprehensive structural model of dispositional counter‐empathy, specifying schadenfreude, gluckschmerz, and affective sadism as cardinal dimensions of counter‐empathy. We also develop and validate a multidimensional model of dispositional counter‐empathy, the VICE, which includes the first measures of dispositional gluckschmerz and affective sadism. This model and measure can help expand the study of vicarious emotion beyond empathy, to consider incongruent responses to others' emotions. This model can also help to more clearly situate prior research on counter‐empathic constructs within the broader structure of counter‐empathy to better understand these constructs and guide future research.

## Author Contributions

**Jake R. Siamro:** writing – original draft, review and editing, formal analysis, data curation, conceptualization. **Christian H. Jordan:** writing – review and editing, supervision, conceptualization.

## Ethics Statement

All studies contained were approved by the Research Ethics Board of Wilfrid Laurier University (Ethics approval numbers: 8571, 8733, and 9015).

## Conflicts of Interest

The authors declare no conflicts of interest.

## Supporting information


**Appendix S1:** jopy70023‐sup‐0001‐Supinfo1.docx.

**Data S1:** jopy70023‐sup‐0002‐DataS1.xlsx.

## Data Availability

The data that support the findings of these studies, the syntax, study materials, and supplemental materials are available on OSF.
