# Supplementary material for: Disentangling Counter‐Empathy: Developing a Three‐Dimensional Model and Measure of Dispositional Counter‐Empathy
Source: J Pers. 2025 Oct 7;94(4):584–601. doi: 10.1111/jopy.70023 (PMC13359305; doi:10.1111/jopy.70023)
Supplement: Supplementary file 1 — Appendix S1: jopy70023‐sup‐0001‐Supinfo1.docx. [file JOPY-94-584-s002.docx]

**Supplemental Materials**

## Supplemental Table 1

*Demographic Comparison Between Collected Sample and Census-Based Targets (Study1b)*

| Variable | Census-Based Target | | Recruited Sample | |
| --- | --- | --- | --- | --- |
|  | *n* | % | *n* | % |
| Gender |  |  |  |  |
| Male | 225 | 50.00 | 225 | 50.00 |
| Female | 225 | 50.00 | 225 | 50.00 |
| Age |  |  |  |  |
| 18-29 | 90 | 20.00 | 91 | 20.22 |
| 30-44 | 180 | 40.00 | 184 | 40.88 |
| 45-59 | 90 | 20.00 | 90 | 20.00 |
| 60-99 | 90 | 20.00 | 85 | 18.88 |
| Race |  |  |  |  |
| White | 405 | 90.00 | 405 | 90.00 |
| Black or African American | 45 | 10.00 | 45 | 10.00 |
| Ethnicity |  |  |  |  |
| Hispanic, Latino, or Spanish Origin | 45 | 10.00 | 38 | 8.44 |
| Not Hispanic, Latino, or Spanish Origin | 405 | 90.00 | 412 | 91.56 |

*Note.* Age targets differ between Study 1b and Study 2 due to a glitch on the CloudResearch Connect website.

**Study 1a and Study 1b Supplemental Results**

Data from Study 1a and 1b were analyzed concurrently to examine cross-sample replication. Bartlett’s test of sphericity was significant in sample 1a (χ^2^(7140) = 28161.89, *p*<.001) and sample 1b (χ^2^(7140) = 43290.15, *p*<.001), indicating the items are sufficiently correlated to conduct factor analyses. The Kaiser-Meyer-Olkin Measure of sampling adequacy was above the recommended threshold of .60 for sample 1a (KMO = .935) and 1b (KMO = .962), indicating the items’ common variance is not abundant (McCrosky & Young, 1979; Pett et al., 2003; Tabachnick & Fidell, 2007). A majority of the items in sample 1a (91.67%) and 1b (93.33%) had communalities greater than .40, which is common in the social sciences (Costello & Osborne, 2005). These findings suggest both samples are well-suited for factor analysis (Carpenter, 2018).

##

## Supplemental Figure 1

*Scree Plot of Factor Analysis, Parallel Analysis and MAP Analysis (Study 1a)*


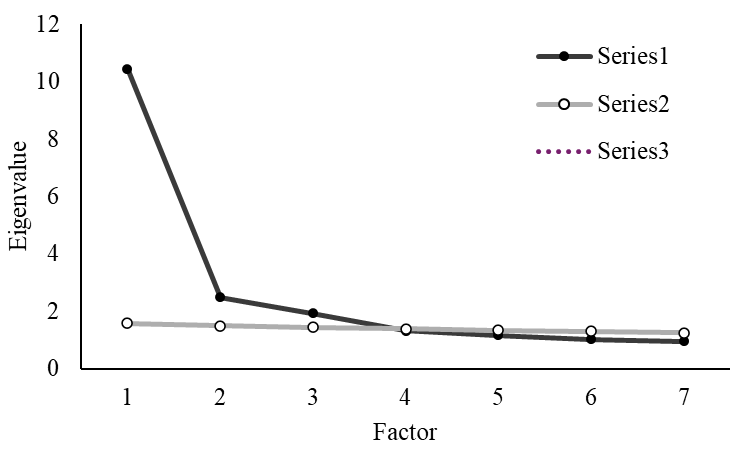


## Supplemental Figure 2

*Scree Plot of Factor Analysis, Parallel Analysis, and MAP Analysis (Study 1b)*


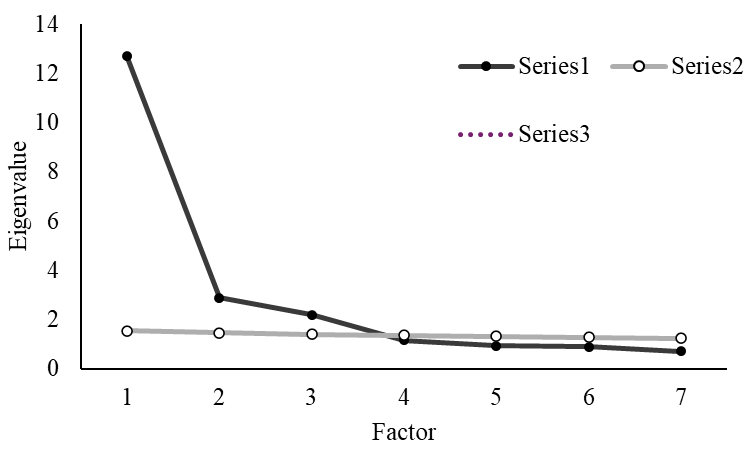


##

## Supplemental Table 2

*Oblique Promax Rotated Factor Loadings of a Principal Axis Factoring Analysis of the 30-Item Various Indices of Counter-Empathy (Study 1 and Study 1b)*

|  | Study 1a | | | | Study 1b | | |
| --- | --- | --- | --- | --- | --- | --- | --- |
|  | Schadenfreude | | Gluckschmerz | Affective Sadism | Schadenfreude | Gluckschmerz | Affective Sadism |
| 1 | | **0.68** | 0.07 | -0.10 | **0.67** | 0.13 | -0.12 |
| 2 | | **0.63** | 0.09 | -0.01 | **0.59** | 0.15 | 0.03 |
| 3 | | **0.56** | 0.07 | -0.03 | **0.78** | -0.11 | -0.04 |
| 4 | | **0.70** | 0.10 | -0.07 | **0.72** | 0.10 | -0.07 |
| 5 | | **0.58** | -0.05 | 0.12 | **0.66** | 0.02 | 0.06 |
| 6 | | **0.55** | -0.12 | -0.08 | **0.70** | -0.11 | -0.17 |
| 7 | | **0.40** | -0.14 | 0.20 | **0.57** | -0.10 | 0.11 |
| 8 | | **0.44** | -0.13 | 0.18 | **0.52** | 0.00 | 0.11 |
| 9 | | **0.43** | 0.04 | 0.20 | **0.42** | 0.05 | 0.12 |
| 10 | | **0.43** | 0.11 | 0.17 | **0.59** | 0.06 | 0.14 |
| 11 | | 0.01 | **0.54** | -0.03 | 0.01 | **0.64** | 0.09 |
| 12 | | 0.08 | **0.72** | -0.09 | 0.04 | **0.69** | 0.03 |
| 13 | | 0.10 | **0.67** | -0.02 | 0.16 | **0.74** | -0.07 |
| 14 | | 0.03 | **0.76** | -0.05 | -0.08 | **0.89** | -0.08 |
| 15 | | -0.02 | **0.55** | 0.22 | -0.03 | **0.72** | 0.08 |
| 16 | | -0.17 | **0.73** | 0.08 | -0.10 | **0.77** | 0.12 |
| 17 | | -0.10 | **0.89** | -0.16 | 0.01 | **0.90** | -0.11 |
| 18 | | -0.05 | **0.69** | 0.13 | -0.01 | **0.71** | -0.06 |
| 19 | | -0.02 | **0.80** | 0.05 | -0.08 | **0.79** | 0.15 |
| 20 | | 0.19 | **0.51** | 0.05 | 0.20 | **0.45** | 0.07 |
| 21 | | 0.12 | 0.05 | **0.48** | -0.01 | -0.01 | **0.76** |
| 22 | | 0.15 | -0.09 | **0.34** | -0.16 | 0.03 | **0.74** |
| 23 | | 0.12 | -0.10 | **0.48** | -0.04 | -0.03 | **0.79** |
| 24 | | 0.25 | 0.02 | **0.45** | 0.07 | 0.08 | **0.72** |
| 25 | | -0.05 | -0.05 | **0.76** | -0.05 | -0.03 | **0.86** |
| 26 | | 0.02 | 0.10 | **0.64** | 0.01 | 0.15 | **0.63** |
| 27 | | -0.06 | -0.01 | **0.87** | 0.03 | 0.05 | **0.82** |
| 28 | | -0.09 | 0.12 | **0.75** | 0.06 | -0.07 | **0.82** |
| 29 | | -0.02 | -0.07 | **0.82** | 0.04 | 0.01 | **0.82** |
| 30 | | -0.03 | 0.13 | **0.70** | 0.04 | -0.05 | **0.89** |

*Note.* Bolded coefficients represent the highest loading for a given scale.

## Supplemental Table 3

*Demographic Comparison Between Collected Sample and Census-Based Targets (Study 2)*

| Variable | Census-Based Target | | Recruited Sample | |
| --- | --- | --- | --- | --- |
|  | *n* | % | *n* | % |
| Gender |  |  |  |  |
| Male | 225 | 50.00 | 225 | 50.00 |
| Female | 225 | 50.00 | 225 | 50.00 |
| Age |  |  |  |  |
| 18-29 | 90 | 20.00 | 93 | 20.67 |
| 30-44 | 90 | 20.00 | 95 | 21.11 |
| 45-59 | 180 | 40.00 | 181 | 40.22 |
| 60-99 | 90 | 20.00 | 81 | 18.00 |
| Race |  |  |  |  |
| White | 405 | 90.00 | 405 | 90.00 |
| Black or African American | 45 | 10.00 | 45 | 10.00 |
| Ethnicity |  |  |  |  |
| Hispanic, Latino, or Spanish Origin | 45 | 10.00 | 45 | 10.00 |
| Not Hispanic, Latino, or Spanish Origin | 405 | 90.00 | 405 | 90.00 |

*Note.* Age targets differ between Study 1b and Study 2 due to a glitch on the CloudResearch Connect website.

## Supplemental Table 4

*Correlations between the Various Indices of Counter-Empathy (VICE) and the Four-Factor Model of Antagonism in (Study 2)*

|  | Various Indices of Counter-Empathy (VICE) | | | | |
| --- | --- | --- | --- | --- | --- |
| Variable | | Total | Schadenfreude | Gluckschmerz | Affective Sadism |
| FFM ATM | |  |  |  |  |
| Antagonism | | .57** | .49** | .40** | .51** |
| Emotional Stability | | -.18** | -.08 | -.32** | .01 |
| Impulsivity | | .35** | .33** | .23** | .29** |
| Agency | | -.11* | -.13* | -.10 | .01 |

*Note.* * indicates *p* ≤ 0.05. ** indicates *p* ≤ 0.001.

## Supplemental Table 5

*Factor Loadings at the Fifth Level of the Bass-Ackward Factor Analysis (Study 2)*

| Variable | F5.1 | F5.2 | F5.3 | F5.4 | F5.5 |
| --- | --- | --- | --- | --- | --- |
| VICE_1 | **0.55** | 0.01 | -0.05 | 0.09 | -0.02 |
| VICE_2 | **0.45** | 0.00 | 0.05 | 0.21 | 0.00 |
| VICE_3 | **0.79** | 0.02 | -0.08 | -0.01 | -0.04 |
| VICE_4 | **0.56** | -0.01 | 0.07 | 0.12 | 0.00 |
| VICE_5 | **0.66** | 0.09 | -0.01 | 0.07 | -0.06 |
| VICE_6 | **0.86** | -0.14 | -0.14 | -0.04 | -0.01 |
| VICE_7 | **0.59** | -0.02 | -0.06 | 0.08 | -0.05 |
| VICE_8 | **0.65** | 0.01 | -0.21 | 0.16 | -0.06 |
| VICE_9 | **0.38** | 0.30 | 0.09 | -0.07 | -0.05 |
| VICE_10 | **0.75** | -0.01 | 0.04 | 0.02 | -0.07 |
| VICE_11 | -0.06 | **0.79** | -0.02 | 0.05 | -0.10 |
| VICE_12 | -0.02 | **0.82** | -0.18 | 0.11 | -0.06 |
| VICE_13 | -0.04 | **0.85** | -0.09 | -0.01 | -0.06 |
| VICE_14 | -0.08 | **0.87** | -0.02 | 0.02 | -0.03 |
| VICE_15 | -0.08 | **0.78** | -0.12 | 0.24 | 0.01 |
| VICE_16 | -0.05 | **0.78** | -0.05 | 0.21 | -0.16 |
| VICE_18 | -0.11 | **0.81** | -0.07 | 0.17 | -0.03 |
| VICE_20 | -0.04 | **0.46** | -0.13 | 0.21 | 0.28 |
| VICE_21 | 0.27 | 0.15 | -0.13 | **0.54** | 0.03 |
| VICE_22 | 0.03 | 0.11 | -0.19 | **0.82** | 0.00 |
| VICE_23 | 0.04 | 0.18 | -0.10 | **0.76** | -0.09 |
| VICE_24 | 0.25 | 0.11 | 0.12 | **0.47** | -0.04 |
| VICE_25 | -0.06 | 0.10 | 0.11 | **0.73** | -0.04 |
| VICE_26 | 0.04 | 0.25 | -0.04 | **0.69** | -0.10 |
| VICE_27 | 0.06 | 0.39 | -0.11 | **0.63** | -0.08 |
| VICE_29 | -0.09 | 0.33 | 0.05 | **0.67** | -0.02 |
| VICE_30 | 0.10 | 0.26 | -0.05 | **0.57** | 0.01 |
| ACME_2 | 0.08 | 0.14 | **0.51** | 0.14 | -0.08 |
| ACME_5 | 0.25 | 0.13 | **0.44** | 0.11 | -0.08 |
| ACME_6 | 0.09 | 0.04 | 0.29 | **0.38** | -0.09 |
| ACME_10 | -0.24 | **0.47** | 0.17 | 0.22 | -0.01 |
| ACME_11 | 0.07 | **0.40** | 0.16 | 0.14 | -0.04 |
| ACME_18 | 0.03 | -0.01 | **0.52** | 0.12 | -0.03 |
| ACME_19 | 0.11 | 0.07 | **0.52** | 0.09 | -0.04 |
| ACME_20 | 0.12 | -0.04 | **0.47** | 0.22 | -0.06 |
| ACME_21 | 0.04 | 0.01 | **0.50** | 0.20 | -0.07 |
| ACME_26 | **0.63** | 0.07 | 0.24 | -0.11 | -0.06 |
| ACME_27 | 0.10 | 0.09 | **0.58** | -0.19 | 0.10 |
| ACME_36 | 0.30 | 0.11 | **0.38** | -0.03 | -0.02 |
| BMES_1 | -0.07 | 0.37 | -0.03 | -0.02 | **0.58** |
| BMES_2 | -0.12 | **0.42** | 0.23 | 0.04 | 0.20 |
| BMES_3 | -0.15 | **0.63** | 0.26 | -0.10 | 0.12 |
| BMES_4 | -0.14 | **0.55** | 0.24 | -0.10 | 0.16 |
| BMES_5 | -0.11 | **0.68** | 0.13 | 0.00 | 0.17 |
| CRY_1R | **0.77** | -0.14 | -0.10 | -0.10 | -0.02 |
| CRY_5R | **0.70** | -0.10 | -0.04 | -0.07 | -0.10 |
| CRY_6 | **0.74** | -0.13 | -0.05 | -0.03 | 0.02 |
| CRY_7 | 0.15 | **0.32** | 0.12 | 0.13 | 0.08 |
| CRY_8R | **0.38** | 0.10 | 0.15 | 0.10 | -0.04 |
| CRY_9R | **0.28** | 0.11 | 0.11 | 0.20 | 0.02 |
| CRY_10 | 0.27 | **0.42** | 0.02 | -0.07 | 0.10 |
| CRY_11R | **0.36** | 0.23 | 0.20 | -0.03 | 0.02 |
| CRY_12 | **0.38** | 0.11 | -0.04 | 0.19 | 0.04 |
| KZ_1 | **0.51** | 0.28 | 0.19 | -0.28 | 0.10 |
| KZ_2 | **0.46** | 0.19 | 0.16 | -0.29 | 0.19 |
| KZ_3 | 0.08 | **0.42** | 0.36 | -0.03 | 0.04 |
| KZ_4 | 0.28 | **0.64** | 0.02 | -0.17 | 0.07 |
| KZ_5 | 0.23 | **0.04** | 0.00 | 0.03 | 0.25 |
| KZ_6 | 0.03 | **0.53** | 0.10 | 0.00 | 0.13 |
| SSIS_1 | 0.05 | 0.01 | **0.62** | 0.15 | -0.07 |
| SSIS_2 | -0.18 | -0.03 | **0.81** | 0.07 | -0.07 |
| SSIS_3 | -0.11 | 0.00 | **0.86** | 0.07 | -0.16 |
| SSIS_4 | -0.11 | -0.07 | **0.89** | -0.05 | -0.11 |
| SSIS_5 | 0.01 | 0.00 | **0.51** | -0.05 | 0.00 |
| SSIS_6 | -0.05 | 0.10 | **0.79** | -0.23 | -0.02 |
| SSIS_7 | -0.11 | -0.03 | **0.74** | -0.08 | -0.04 |
| SSIS_8R | 0.02 | 0.01 | **0.37** | 0.01 | -0.01 |
| SSIS_9 | 0.12 | 0.05 | **0.49** | 0.01 | 0.01 |
| SSIS_10 | 0.03 | 0.11 | **0.61** | -0.23 | 0.08 |
| TP_2 | 0.27 | 0.22 | -0.08 | -0.07 | **0.45** |
| TP_3 | 0.00 | 0.01 | -0.02 | -0.11 | **0.74** |
| TP_5 | -0.07 | 0.04 | -0.11 | 0.22 | **0.45** |
| TP_7 | 0.07 | -0.06 | 0.05 | -0.06 | **0.55** |
| TP_8 | -0.03 | -0.02 | -0.18 | 0.00 | **0.83** |
| TP_10 | -0.01 | 0.10 | -0.11 | 0.21 | **0.59** |
| TP_13 | -0.07 | 0.12 | -0.09 | 0.18 | **0.58** |
| TP_15 | 0.09 | 0.00 | -0.08 | 0.08 | **0.62** |
| TP_18 | -0.06 | -0.04 | 0.02 | -0.10 | **0.82** |
| TP_19 | -0.13 | -0.11 | 0.05 | -0.01 | **0.77** |
| VAST_1 | **0.47** | -0.38 | 0.10 | 0.24 | 0.17 |
| VAST_2 | 0.19 | -0.27 | 0.16 | **0.34** | 0.10 |
| VAST_3 | 0.37 | **-0.45** | 0.13 | 0.28 | 0.15 |
| VAST_5 | **0.34** | -0.08 | -0.10 | 0.31 | 0.15 |
| VAST_7 | **0.45** | -0.26 | 0.20 | 0.23 | 0.06 |
| VAST_8 | -0.11 | -0.17 | 0.46 | **0.52** | 0.09 |
| VAST_9R | 0.21 | 0.04 | **0.44** | -0.09 | -0.01 |
| VAST_10 | **0.27** | -0.01 | 0.17 | 0.11 | 0.02 |
| VAST_11 | -0.22 | -0.08 | 0.51 | **0.37** | 0.12 |
| VAST_12 | -0.03 | -0.11 | **0.56** | 0.17 | 0.08 |
| VAST_13 | -0.10 | -0.06 | **0.63** | 0.31 | 0.06 |
| VAST_14 | 0.05 | -0.13 | **0.46** | 0.36 | 0.11 |
| VAST_16 | -0.16 | -0.22 | 0.22 | **0.51** | 0.23 |

*Note.* F5.1 Schadenfreude. F5.2 Gluckschmerz. F5.3 Everyday Sadism. F5.4 Affective Sadism. F5.5 Favor Fall.

## Supplemental Table 6

*Correlations Between Level 5 Bass-Ackward Factors (Study 2)*

| Variable | F5.1 | F5.2 | F5.3 | F5.4 |
| --- | --- | --- | --- | --- |
| F5.1 Schadenfreude | - |  |  |  |
| F5.2 Gluckschmerz | .51 | - |  |  |
| F5.3 Everyday Sadism | .68 | .58 | - |  |
| F5.4 Affective Sadism | .44 | .45 | .54 | - |
| F5.5 Favour Fall | .57 | .58 | .51 | .24 |

*Note.* All correlations are significant where *p* ≤ 0.001.

**Study 3b (Undergraduate Sample) Supplemental Results**

***Participants***

Canadian undergraduates (*N* = 335) participated for partial course credit. However, we deliberately oversampled in anticipation of data exclusions. Using preregistered criteria, participants were excluded for having too much missing data (> 10% of items; *n* = 2), reporting their data should not be used (*n* = 3), inattentive responding (*n* = 17), responding overly quickly (one third of the median completion time; *n* = 3), or overly slowly (> 1.25 hours; *n* = 16). The final sample consisted of 294 participants (11.2% male, 85.7% female, 2.4% non-binary, and .7% other) ranging in age from 17 to 51 (*M* = 19.78, *SD* = 3.45) and identifying their ethnicity as: White (70.1%), South Asian (9.5%), Middle Eastern (3.4%), East Asian (3.1%), African (2.0%), Latin, Central, or South American (2.0%), Caribbean (1.7%), Indigenous (.3%), and other (7.8%).

***Results***

VICE items were presented in an interspersed order instead of in the typical order, which may have greatly inflated their intercorrelations relative to other samples (VICE-schadenfreude/VICE-gluckschmerz *r* = .76; VICE-schadenfreude/VICE-affective sadism *r* = .76; VICE-gluckschmerz/VICE-affective sadism *r* = .82). VICE-schadenfreude predicted positive affect in response to these vignettes (*r* = .28), as did VICE-gluckschmerz (*r* = .21) and VICE-affective sadism (*r* = .31). The relation with VICE-schadenfreude remained significant when controlling cognitive empathy, β = .08, *t*(291) = 3.81, *p* < .001, and affective empathy, β = .09, *t*(291) = 4.44, *p* < .001. Which was also similar to that observed for other schadenfreude measures (CSS *r* = .26; SS-Total r = .31; SS-Benign *r* = .15; SS-Malicious *r* = .37).

**Study 3a Target Morality Results**

***Vignettes***

In addition to the vignettes described in Study 3a, the morality of the target (i.e., immoral or control) was manipulated. As described in Study 3a, each vignette involved witnessing someone experiencing a fortunate (e.g., receiving a prestigious scholarship) or unfortunate (e.g., falling flat at a comedy night) event. Additionally, four scenarios depicted an immoral target (e.g., a thief), and four scenarios depicted a neutral target (e.g., an individual you have seen but never spoken to). Due to an error by the researcher, three good fortune vignettes depicted an immoral target, and one good fortune vignette depicted a neutral target. Deservingness was conceptualized as a justice-based variable where some degree of personal responsibility was attributed to the individual for their outcome due to their immoral character. Therefore, by manipulating the morality of the target individual, we predicted that fortunate outcomes would be seen as undeserved and unfortunate outcomes would be seen as deserved.

***Results***

**The Effect of Target Morality on Affect.** To test the degree to which manipulating morality elicited an increased counter-empathic response in response to good and bad fortune, a series of paired-samples t-tests were run. As predicted, positive affect scores in the immoral condition for the misfortune scenarios (*M* = 2.55, *SD* = .99) were significantly higher than in the control condition (*M* = 1.25, *SD* = .52), *t*(377) = 23.20, *p* < .001. Additionally, as expected, negative affect scores in the immoral condition for the good fortune scenarios (*M* = 3.17, *SD* = .93) were significantly higher than in the control condition (*M* = 1.29, *SD* = .65), *t*(377) = 35.17, *p* < .001.

To better understand the relationship between immorality and trait counter-empathy, a series of within-subjects moderation analyses were run. We hypothesized that individuals with low trait schadenfreude scores would have significantly higher positive affect in the immoral condition compared to the neutral condition. The analysis revealed that responses to the immoral condition good fortune vignettes were generally less positive than in the neutral condition (*F*(1, 376) = 55.07, *p* < .001*,* $\eta_{p}^{2}$ = .13). Schadenfreude did not significantly predict positive affect (*F*(1, 376) = 2.55, *p* = .111*,* $\eta_{p}^{2}$ = .01). Contrary to our hypothesis, the predicted interaction was not significant (*F*(1, 376) = .02, *p* = .883*,* $\eta_{p}^{2}$ = .00).

We also hypothesized that individuals with low trait gluckschmerz scores would have significantly higher negative affect in the immoral condition compared to the neutral condition. We found that responses to the immoral condition misfortune vignettes were generally more negative than in the neutral condition (*F*(1, 376) = 235.65, *p* < .001*,* $\eta_{p}^{2}$ = .39). Gluckschmerz was also a significant predictor of negative affect (*F*(1, 376) = 25.61, *p* < .001*,* $\eta_{p}^{2}$ = .06). Contrary to our hypothesis, the predicted interaction was not significant (*F*(1, 376) = .01, *p* = .913*,* $\eta_{p}^{2}$ = .00).

##

## Supplemental Table 7

*Correlations between Counter-Empathy, Empathy and Fortune Vignette Reactions (Study 3b)*

|  | Positive Affect | Empathic Affect |
| --- | --- | --- |
| VICE Total | .55 | -.16 |
| VICE Schadenfreude | .38 | -.21 |
| VICE Gluckschmerz | .41 | -.08 |
| VICE Affective Sadism | .55 | -.07 |
| CSS Schadenfreude | .42 | -.18 |
| SS Benign Schadenfreude | .21 | -.15 |
| SS Malicious Schadenfreude | .43 | -.22 |
| SS Total Schadenfreude | .36 | -.21 |
| VAST Direct Sadism | .31 | -.15 |
| VAST Vicarious Sadism | .42 | -.16 |
| QCAE Cognitive Empathy | -.17 | .31 |
| QCAE Affective Empathy | -.18 | .32 |

*Note.* All correlations above a value of |.10| are significant where *p* ≤ 0.05.

## Supplemental Table 8

*Hierarchical Regression Predicting Aggression with Empathy and Counter-Empathy (Study 2)*

| Variable | Δ*R*^2^ | *B* | *SE B* | *β* | 95% CI | |
| --- | --- | --- | --- | --- | --- | --- |
| Step 1 | .11** |  |  |  |  |  |
| IRI-Empathy |  | -.10 | .06 | -.11 | -.21 | .01 |
| ACME-Empathy |  | -.32 | .07 | -.26** | -.46 | -.18 |
| Step 2 | 24** |  |  |  |  |  |
| IRI-Empathy |  | -.01 | .05 | -.01 | -.11 | .10 |
| ACME-Empathy |  | -.19 | .07 | -.17* | -.34 | -.07 |
| VICE-Schadenfreude |  | .19 | .03 | .31** | .13 | .25 |
| VICE-Gluckschmerz |  | .11 | .03 | .17** | .05 | .18 |

*Note.* * indicates *p* ≤ 0.05. ** indicates *p* ≤ 0.001.
